# A Robust Topology-Based Algorithm for Gene Expression Profiling

**DOI:** 10.5402/2012/381023

**Published:** 2012-11-11

**Authors:** Lars Seemann, Jason Shulman, Gemunu H. Gunaratne

**Affiliations:** ^1^Department of Physics, University of Houston, Houston, TX 77204, USA; ^2^Department of Physics, Richard Stockton College of New Jersey, Pomona, NJ 08240, USA

## Abstract

Early and accurate diagnoses of cancer can significantly improve the design of personalized therapy and enhance the success of therapeutic interventions. Histopathological approaches, which rely on microscopic examinations of malignant tissue, are not conducive to timely diagnoses. High throughput genomics offers a possible new classification of cancer subtypes. Unfortunately, most clustering algorithms have not been proven sufficiently robust. We propose a novel approach that relies on the use of statistical invariants and persistent homology, one of the most exciting recent developments in topology. It identifies a sufficient but compact set of genes for the analysis as well as a core group of tightly correlated patient samples for each subtype. Partitioning occurs hierarchically and allows for the identification of genetically similar subtypes. We analyzed the gene expression profiles of 202 tumors of the brain cancer glioblastoma multiforme (GBM) given at the Cancer Genome Atlas (TCGA) site. We identify core patient groups associated with the classical, mesenchymal, and proneural subtypes of GBM. In our analysis, the neural subtype consists of several small groups rather than a single component. A subtype prediction model is introduced which partitions tumors in a manner consistent with clustering algorithms but requires the genetic signature of only 59 genes.

## 1. Introduction

Cancers in many tissues are heterogeneous, and the efficacy of therapeutic interventions depends on the specific subtype of the malignancy. Hence, early and accurate identification of the cancer subtype is critical in designing an effective personalized therapy. Current methods for assessment rely on microscopic examinations of the malignant tissue for previously established histopathological abnormalities. Unfortunately, such features may not be apparent during early stages of the disease and moreover, differentiating between abnormalities in distinct cancer subtypes can be challenging. Recent advances in high-throughput genomics offer an exciting new alternative for early and reliable cancer prognosis. Mutations that underlie a malignancy modify the levels of many genes within a cell; the goal of gene expression profiling is to define a signature for each cancer subtype through statistically significant up-/downregulation of a panel of genes. The National Institutes for Health, through the Cancer Genome Atlas (TCGA) [1, 2], will aid this effort by establishing large sets of genomic data on human cancers in at least 20 tissues [3–8].

**Figure 1:**
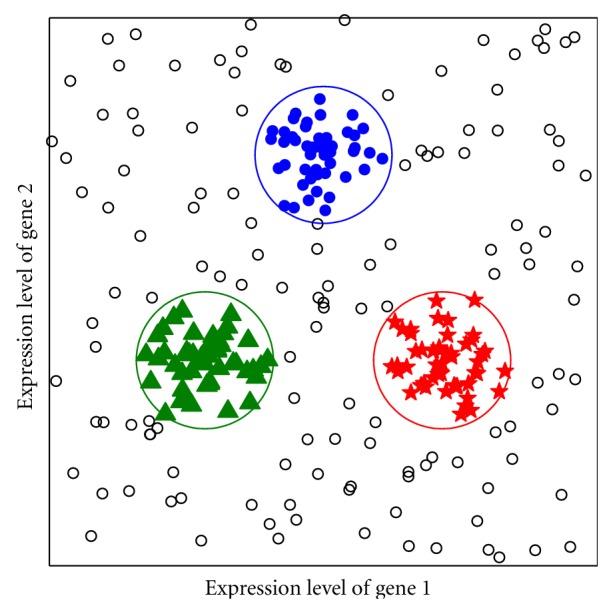
The layout of the point cloud associated with a “schematic” cancer represented by expression levels *X*_1_ and *X*_2_ of two genes. The point cloud is distributed randomly but contains three closely packed “pockets,” each of which is assumed to consist of “core” samples of a specific subtype of the malignancy.

The premise behind TCGA is that statistically significant changes in gene expression levels due to malignant mutations can be placed in a few groups associated with subtypes, and that unsupervised (or semisupervised) clustering algorithms can be used to uncover these partitions. This is illustrated through a schematic “malignancy” that can be partitioned using the expression levels of two genes. In this schematic, each patient sample is represented by a point on a plane, see Figure 1. The basic observation is that, while the patient samples are distributed over a broad range, there are “pockets” of high concentration, which can be identified using traditional clustering methods. The members of the pocket define the “core” samples of a cancer subtype. However, the presence of significant levels of noise in genomic data makes the partitioning a nontrivial task. The variability is due to both the subject dependence of the expression levels and to imperfections in microarray technology. Unfortunately, costs associated with microarray experiments prohibit the use of a large set of replicates to reduce the effective error rates.

Progress has been made in spite of these difficulties. One focus of TGCA has been *glioblastoma multiforme* (GBM), the most common and aggressive form of brain cancer [9, 10]. TCGA provides the expression levels of 11861 genes in 200 GBM and 2 normal brain samples [1, 2]. Reference [1] identifies 1740 genes with consistent expression across Affymetrix HuEx, Affymetrix U133A (Affymetrix, Santa Clara, CA, USA), and Agilent 244K Common Genomic hybridization arrays (Agilent Technologies, Santa Clara, USA), to be used for the subgrouping. They search for common partitions under sampling of genes and patients. The resulting *consensus clustering* [11] yields four robust clusters whose class boundaries are statistically significant [1]. 173 “core” representatives of the four groups were identified, and an 840-gene signature was determined on the basis of lowest cross-validation and prediction error [1]. This genomic partitioning of the 173 core samples was found to be consistent with the grouping into the four known subtypes classical, mesenchymal, proneural, and neural of glioblastoma.

In this work, we introduce a new algorithm for gene expression profiling, which is illustrated through an application to GBM. This approach avoids several difficulties associated with clustering algorithms commonly adopted to partition large sets of genomic data. It provides robust partitions of patients and identifies a compact set of genes used to distinguish the clusters. In particular, for GBM, the clusters of patients are sharply defined leading us to establish the quintessential genetic profile for the subtypes. Further, the newly identified set of genes will allow tumors of new patients to be subtyped quickly after diagnosis, which perhaps could lead to a more personalized and successful treatment regimen. Most of these genes have not been previously implicated in brain cancer or cancer in general; they may be unidentified members of the cancer network.

Unfortunately, significant structural variation in tumor samples renders it impossible to determine subtype by histological methods [3]. Thus, we are unable to have independent corroboration of our results and are left to compare our calculation with the clustering analysis of the TGCA data.

## 2. Approach

Our algorithm requires the panel of genes used for clustering to be predetermined. Due to stochasticity, too few genes are unlikely to provide a reliable classifier for cancer subtypes. The choice of an excessively large set of genes is likely to be counter productive as well, at least in part due to contamination from the many genes that are irrelevant for the comparison. A large gene set will cause a another well-known difficulty, namely, that of partitioning a (relatively) small number of objects in a high dimensional space [12]. Thus, it is important to preselect a sufficient yet compact set of genes for clustering. This issue is resolved using a statistical invariant and persistent homology [13–15], one of the most exciting recent developments in topology. A unique feature of our approach is the hierarchical partitioning of patient samples. The samples are repeatedly bisected until further partitioning is not possible. This yields the appropriate number of clusters and provides information on the genetic proximity of the subtypes. Additionally, refinements to the panel of genes used in the partitioning are possible through repeated application of the method.

Clustering algorithms require a notion of proximity between patient samples, see Figure 1. Herein lies a second difficulty. There is no natural “genomic distance” between two samples, although the correlation distance (defined below) is used in most studies. Hence, it is desirable to use a topological approach (such as persistent homology that is adapted here) for partitioning, rather than one which relies on computation of metrics such as eigenvalues. This is likely the reason why hierarchical clustering [16] typically provides a more robust partitioning than spectral clustering [17–20] or those based on principal components analysis [21]. We use persistent homology for partitioning.

The selection of the optimal number of partitions is another nontrivial task in clustering [11]. *Empirical cumulative distribution functions*, which quantify the proximity of a given clustering to a “perfect” grouping (where the membership of each object is unambiguous) is one of the more successful approaches to address the problem in genomic data [11]. We do not predetermine the number of partitions. At each stage, the patient samples are bisected. If a partition requires further subgrouping, the algorithm is applied recursively on it.

Next, we wish to introduce a prediction model to assign the cancer subtype. To this end, we randomly select 20 (“test” samples) of the 202 patient samples which are only to be used for validation. The remaining 182 patient samples form the “training set.” We have chosen to implement each bisection using 30 genes. (The algorithm is “robust” in the sense that the patient subgroups obtained from the algorithm do not depend on the number of genes selected for the panel.) One partition contains 60 samples, 27 of which labeled classical by TCGA and 28 of which labeled mesenchymal. The second partition of 44 elements contains 37 proneural samples. Bisection of the first group is, once again, performed using 30 genes. Its partitions contain 18 (17 classical) and 14 (all mesenchymal) patient samples. Thus, our method identifies the three primary clusters of GBM. In our calculation, the neural group was not found to be a single, coherent cluster. A total of 59 genes are used for the two bisections. Interestingly, core nodes of the biological network underlying GBM such as *TP53*, *PTEN*, *RB1*, *NF1*, *PIK3R1*, and *PIK3CA* [1, 22] are not in this list.

Finally we use the 59-gene set to assign the cancer subtypes in the 20 test samples, comparing the conclusions with those of the clustering calculation “http://tcga-data.nci.nih.gov/docs/publications/gbm_exp/” [1]. The assessment of the subtype of each test sample is made using the mean of its correlation distance from the corresponding core patient samples. Of the 20 test samples, 15 were in agreement with the clustering algorithm. Five of these were not assigned a group by either our calculation or that of TCGA. Four samples, which were labeled neural, were not provided an assignment by our algorithm.

## 3. Methods

### 3.1. Nondimensionalized Standard Deviation

**Figure 2:**
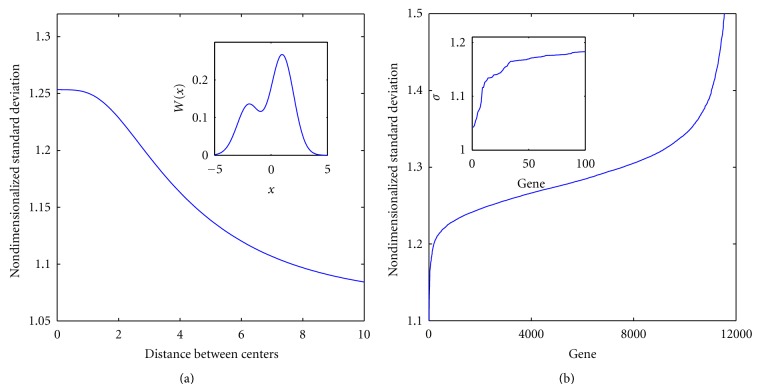
The nondimensionalized standard deviation *σ* is used to identify genes whose expression levels may lie on bimodal distributions. (a) The behavior of *σ* for *W*(*x*) = *η*_1_ *W*_0_(*x*; *a*_1_) + *η*_2_ *W*_0_(*x*; *a*_2_) as a function of *a* = |*a*_2_ − *a*_1_|, where *W*_0_(*x*; *a*_*k*_) is the Gaussian distribution of unit standard deviation centered at *a*_*k*_. In this computations *η*_1_ = 1/3 and *η*_2_ = 1 − *η*_1_. The inset shows the distribution *W*(*x*) when *a* = 3. It appears to contain two statistically distinct components. (b) The values of *σ* for the 11861 genes given at the TGCA site, in increasing order. Approximately 50 genes have values of *σ* significantly smaller than the rest.

A gene can only be useful in differentiating between subtypes of cancer if its expression levels in the subtypes lie on statistically distinct distributions [1, 23]. A necessary (but not sufficient) condition is the bimodality of the corresponding distribution for all patient samples. (Note that, at this stage, we do not know the mean values, variances, or the membership of the putative subgroups. Hence standard statistical tools such as discriminant analysis [24, 25] cannot be used to decide if and how patient samples are to be partitioned.) We propose the use of the *nondimensionalized standard deviation*

(1) $$\begin{array}{c} \sigma =\frac{\sqrt{\mathrm{Var}\;\left(X\right)}}{𝔼\left[\left|X-𝔼\left[X\right]\right|\right]}, \end{array}$$

to select such genes. Here *𝔼*[**X**] represents the expectation value of a random variable **X** and Var (**X**) = *𝔼*[**X** ^2^] − *𝔼*[**X**]^2^ its variance. The definition is inspired by kurtosis, which is a scale-invariant measure of the “width” of a distribution [26]. Unfortunately, it is difficult to estimate the fourth moment reliably from 182 noisy points (gene expression levels) [27]. *σ* is an alternative scale-invariant characterization of a distribution's width. *σ* is larger for distributions with broad tails: for Gaussian and biexponential distributions its values are $\sqrt{\pi /2}$ and $\sqrt{2}$, respectively. Its value for a finite-range characteristic function (which has no tails) is 2/$\sqrt{3}$. Variation of *σ* as a function of the distance between two Gaussian distributions is shown in Figure 2(a), demonstrating that bimodality of a distribution can be inferred from the value of *σ*.

 The gene expression profiles for the 202 patient samples are given at “http://tcga-data.nci.nih.gov/docs/publications/gbm_exp/.” The Table “unifiedScaled.txt” contains mean expression levels (from three replicates) for 11861 genes of each patient sample. The expression levels of each gene are normalized.

Figure 2(b) shows the values of *σ* for the 11861 genes in increasing order. (For each gene, we have discarded the extreme outliers, which are more than 8 standard deviations away from the mean.) A very small fraction of genes have abnormally small values of *σ*, which we associate with multimodal distributions. The eight smallest values of *σ* are found for genes *EIF1AY*,* RPS4Y1, DDX3Y, UTY, USP9Y, JARID1D, ZFY*, and *NLGN4Y*. The homology analysis (see below) shows that these eight genes and *CYorf15B* belong to one group, that is, the correlation distance between these genes is small. However, each gene in this group belongs in the Y-chromosome, and hence the associated partitioning is that between male and female subjects. We eliminate the group from further consideration. Of the remaining genes, we select *N* = 60 that have the smallest values of *σ*. The final partitioning of patients is independent of *N* for choices between 50 and 120, and persistent homology fails to find appropriate partitions when *N* is outside this range. Our assertion of the robustness of the algorithm is a reflection of this fact.

### 3.2. Persistent Homology

Not all of the 60 genes selected using *σ* may be useful for subdividing patient samples. Our second refinement is to search for groups of genes whose expression levels change coherently between patient samples. This coherence can, for example, result from the genes' membership in a subnetwork. For this task, we represent a gene *G*_*n*_ by its expression levels **X**_*n*_ ∈ ℝ^*M*^ in all *M* = 182 patient samples; the 60 genes form a “point cloud.” As is traditional in clustering algorithms, the proximity *d*(**X**_*n*_, **X**_*m*_) between two points **X**_*n*_ and **X**_*m*_ is chosen to be the correlation distance:

(2) $$\begin{array}{c} d\left(X_{n},X_{m}\right)\equiv 1-C\left(X_{n},X_{m}\right)=1-\frac{𝔼\left[X_{n}X_{m}\right]-𝔼\left[X\right]𝔼\left[X_{m}\right]}{\sqrt{\mathrm{Var}\;\left[X_{n}\right]}\sqrt{\mathrm{Var}\;\left[X_{m}\right]}}, \end{array}$$

where *C*(**X**_*n*_, **X**_*m*_) is the Pearson correlation function [28]. We have now converted the search for critical genes to clustering a point cloud. This task could be implemented through algorithms such as hierarchical clustering [16, 29], spectral clustering [20, 30], or community structures [19]. Spectral approaches are highly sensitive to the precise genes chosen for the analysis and to cutoff values, and hierarchical clustering does not provide an unambiguous estimate for the number of partitions.

Our approach relies on *persistent homology* [13–15], a robust topological approach. The components are identified as *simplicial complexes*, which are defined using *simplices*. Consider a point cloud and a set of connections between a subset of pairs of points. A *k*-*simplex* is a set of (*k* + 1) points, each pair of whose vertices is connected. For example, a 1 simplex is a line along with its two vertices, a 2 simplex is a triangle along with its edges and vertices, and a 3 simplex is a tetrahedran along with its faces, edges, and vertices. The *face* of a *k* simplex is a subset which is a simplex of lower order. For example, the vertices, edges, and triangular sides of a tetrahedran are its faces. A *k* *simplicial complex* is an object formed by gluing together faces of a set of simplices each of whose dimension is less than or equal to *k*; the intersection of any two such simplices is required to be a face of both. For example, an object formed by gluing a set of triangles on their edges or vertices, with perhaps some lines and isolated points, is a 2-simplicial complex.

We wish to determine how the point cloud can be partitioned. The question is addressed by constructing a simplicial complex and searching for its disjoint components. First, for a prespecified cutoff radius *r*_*c*_, connect points **X**_*m*_ and **X**_*n*_ if and only if *d*(**X**_*n*_, **X**_*m*_) < *r*_*c*_. The object so formed is a simplicial complex [15]. For sufficiently small *r*_*c*_ no points are connected, and the simplicial complex is the point cloud itself [15]. When *r*_*c*_ is sufficiently large, all pairs of points are connected, and the simplicial complex is a single component.

Simplicial complexes can be characterized using topological invariants (i.e., features that do not change under smooth distortions of space). One of these invariants is the number of disjoint components, which is referred to as the zeroth Betti number (Betti_0_). This is the only feature that is computed in clustering algorithms. Homology theory introduces additional topological invariants of a simplicial complex. The first Betti number, Betti_1_, is the number of connected components with a two-dimensional hole. For example, Betti_1_ for figure-8 is 2. The second Betti number (Betti_2_) is the number of surfaces enclosing a three-dimensional region. Betti_2_ = 1 for the surface of a sphere. The first three Betti numbers for the crust of a bagel are 1, 2, and 1. Betti numbers can provide a more comprehensive invariant characterization of the point cloud than possible through clustering. Whether they will be useful in cancer diagnostics remains to be seen. (Persistent homology has been used successfully to search for recurrent genomic instability in breast cancer [31, 32].)

A partition will be *robust* if the components (of the point cloud) are far apart from each other; consequently, the number of components will remain unchanged for a broad range in *r*_*c*_. A generalization of this statement forms the novel proposal in [13, 14], which associates robust characteristics of the point cloud with “persistent” topological invariants.

**Figure 3:**
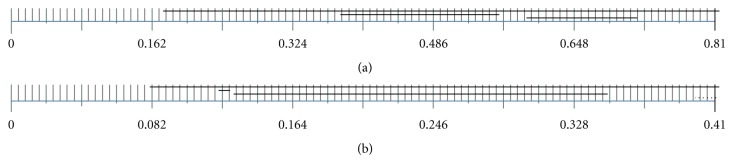
Using persistent homology for partitioning a set of objects. (a) The barcode on the top panel shows groups of genes of size 5 or more as a function of the cutoff radius *r*_*c*_. Each horizontal line represents a component that maintains its identity as *r*_*c*_ is increased. In our analysis, we select the two gene groups *C*_1_′ and *C*_2_′ at *r*_*c*_ ≈ 0.56, just prior to their merging. *C*_1_′ and *C*_2_′ contain 17 and 20 genes, respectively. (b) The bottom panel shows partitions of 10 or more patient samples computed using the expression levels of members of *C*_1_′ and *C*_2_′. The patient samples are bisected at *r*_*c*_ ≈ 0.34 into two partition of sizes 84 and 76. The first group contains patient samples of the classical and mesenchymal subtypes, while the second contains samples of the proneural subtype.

Our computations are implemented using the package *JPlex* (available at “http://comptop.stanford.edu/programs/jplex/index.html”). We note first that if *r*_1_ < *r*_2_, the simpicial complex at cutoff *r*_1_ is contained in that at *r*_2_. The primary construction used in *JPlex* is the “Rips Stream,” which computes the simplices at cutoff radii *r*_*c*_ in a prespecified range, and assigns a “filtration time” for each simplex, when it first appears. “Barcodes,” such as those shown in Figure 3, show how the number of components evolves as *r*_*c*_ is increased. The abscissa is the cutoff radius and each horizontal line represents a component that maintains its identity as *r*_*c*_ increases. It should be noted that the membership of a component may increase with *r*_*c*_. In Figure 3(a), we only show components that contain a minimum of *N*_min_ points. This constraint is imposed by constructing a new rips stream which contains the original simplices; the filtration time associated with each simplex in the new stream is defined to be the maximum of the original filtration time and the time when the component reaches *N*_min_ elements.

The top panel of Figure 3 shows the results of the analysis for the 60 genes with *N*_min_ = 5. We select the two components that persist for the largest range in *r*_*c*_. The membership of the groups is maximized at *r*_*c*_ ≈ 0.56, just prior to their combining into a single component. (The persistence width of the component that begins after *r*_*c*_ = 0.56 decreases as the number *N* of genes used for the analysis increases.) Genes in each component are found using the *JPlex* routine “verticesInEachComponent.” At *r*_*c*_ = 0.56, the components contain 17 and 20 genes, respectively. We could choose all 37 genes for the analysis. However, we find that identical partitioning of the patient samples can be achieved with a smaller number of genes. For the analysis here, we select the first *N*_*g*_ = 15 genes to join each component. This choice is made in order to include a sufficiently inclusive, yet not too large a set of genes for the analysis. The final grouping of patient samples depends only weakly on the choice of *N*_*g*_. The two gene sets are

(3) C1′={UGT8,MBP,C11orf9,MOG,KLK6,RP11−35N6.1,RAB33A,DCX,GPR17,CXorf1,LRRTM4,TMSL8,SN,AP91,ATP10B,LUZP2},C2′={MEOX2,PIPOX,FGFR3,DLC1,PLA2G5, EGFR,GRIK1,ERBB2,PTRF,POSTN,BAIAP3, PDLIM4,KCNF1,EYA4,SPAG4}.

Genes in *C*_1_′ lie on chromosomes 1, 2, 3, 4, 5, 6, 7, 8, 10, 13, 17, 20, and 21. Genes *MEOX2*, *EGFR*, *DCL1*, *EFGR*, *PTRF*, and *EYA4* have the gene ontology (GO) classification “nucleus.” Genes *MEOX2*, *DCL1*, *ERBB2*, *PTRF*, *EYA4*, *SPAG4* have the classification “cytoplasm,” and *FGFR3*, *GRIK1*, *KCNF1* have the classifications “membrane” and “integral to membrane.” Genes in *C*_2_′ belong to chromosomes 1, 2, 4, 5, 6, 9, 11, 15, and the X-chromosome. *C11*or*f9* and *KLK6* have the gene ontology classification “nucleus,” while *DCX*, *KLK6*, and *TMSL8* have the classification “cytoplasm,” and *UGT8*, *C11*or*f9*, *MOG*, *GPR17*, *LRRTM4*, and *ATP10B* have the classification “membrane.”

### 3.3. Partitioning Patient Samples

**Figure 4:**
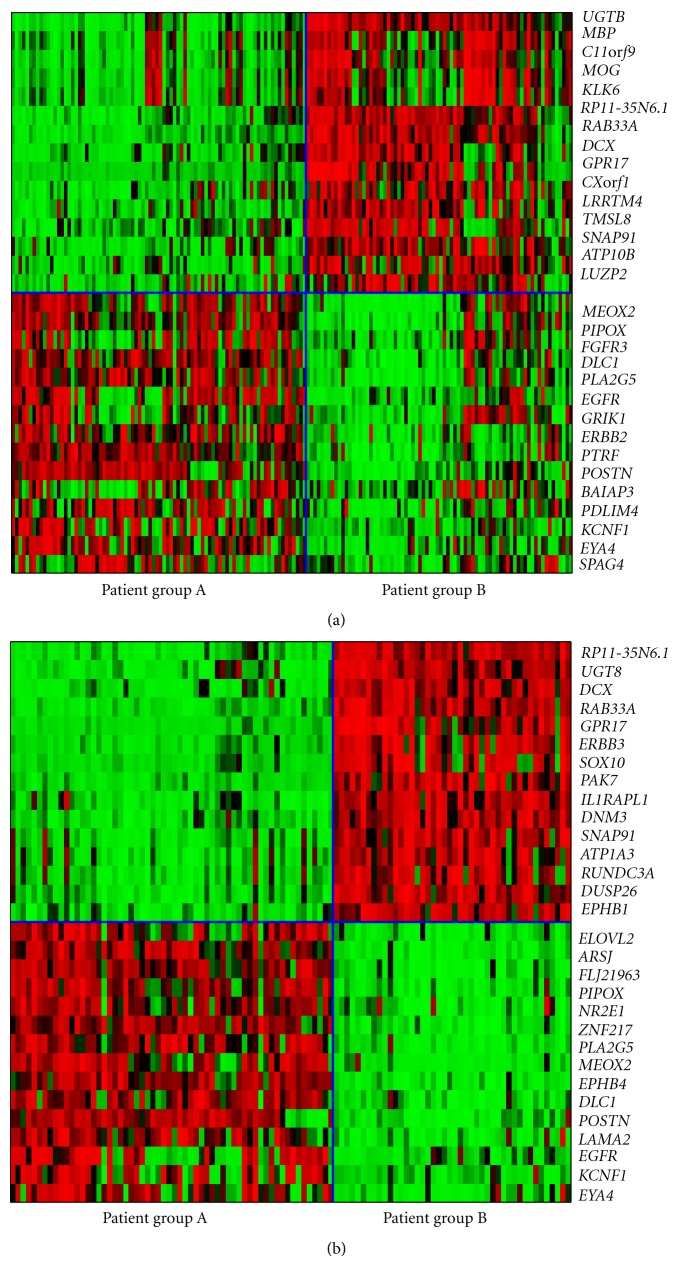
(a) The heat map for the bisection of GBM patient samples with red (resp., green) representing fold inductions higher (resp., lower) than the mean. The horizontal blue line separates the gene groups *C*_1_′ and *C*_2_′, while the vertical line partitions the two components of patient samples. The patient samples are arranged (left to right) in the order they join each component. It is clear that samples joining a component later are less tightly correlated to the group. (b) The heat map for the core patient samples in each component using genes in *C*_1_ and *C*_2_. Component A contains classical and mesenchymal patient samples, while B contains proneural samples.

The 182 patient samples are partitioned using the expression levels of the 30 genes in *C*_1_′ and *C*_2_′. Each sample is represented by the expression levels **Y** of these 30 genes. As before, the distance between two points (patient samples) **Y**_*m*_ and **Y**_*n*_ in ℝ^30^ is defined as the correlation distance. The barcode for the analysis, where we retain only groups of size 10 or larger, is shown in Figure 3(b). Two groups are seen to persist over a significant range in the cutoff radius *r*_*c*_. Since the components grow with *r*_*c*_, we choose *r*_*c*_ ≈ 0.34, in order to maximize their membership. They contains 84 and 76 patient samples, and the heat map [33–35] is shown in Figure 4(a). The patient samples in each group are arranged in the order they appear in the component as *r*_*c*_ is increased. The fold inductions for genes in the left-most samples in each group are significantly closer than those joining the component later. We propose to only include the most coherent patient samples in the “core” group associated with each category, see Figure 1. With this trimming, the membership of the two groups reduces to 60 and 44. The new heat map, Figure 4(b), provides a significantly sharper partitioning than those computed through many previous approaches [1, 2, 11, 12, 36, 37], although the membership in each group is smaller.

The first group of 60 contains 27 patient samples that have been assigned the classical subtype and 28 samples that have been assigned the mesenchymal subtype of GBM by the clustering analysis of TCGA. The remaining 5 samples are not provided an assignment in [1]. 37 of the 44 patient samples in the second group have been assigned to the proneural subtype by TCGA. The rest of the samples in this group are unassigned with the exception of one sample labeled neural and one labeled mesenchymal [1].

### 3.4. Selecting a Gene Set to Differentiate the Subtype

One of our goals is to search for a panel of genes to differentiate between the subtypes of GBM. Thus far, we have used the genes in *C*_1_′ and *C*_2_′ to find two subgroups of the 182 patient sample, and (using the heat map) select the most tightly correlated members in each subgroup. However, when only the 60 and 44 core samples are considered, an alternative gene set may provide a sharper separation of the patient samples. We thus repeat the computations of the last three Subsections, using only the 104 patient samples in the two core groups. The new gene groups are

(4) C1={UGT8,RP11−35N6.1,DCX,RAB33A,GPR17, ERBB3,SOX10,PAK7,IL1RAPL1,DNM3, SNAP91,ATP1A3,RUNDC3A,DUSP26,EPHB1},C2={ELOVL2,ARSJ,FLJ21963,PIPOX,NR2E1, ZNF217,PLA2G5,MEOX2,EPHB4,DCL1, POSTN,LAMA2,EGFR,KCNF1,EYA4}.

Genes in *C*_1_ and *C*_2_ are spread among several chromosomes. Genes *RP11-35N6.1*, *UGT8*, *GPR17*, *IL1RAPL1*, *SNAP91*, and *EPHB1* of *C*_1_ have the GO classifications “membrane” and/or “integral to membrane,” while *DCX*, *RAB33A*, and *DNM3* have the classification “intracellular.” Genes *NR2E1, ZNF217, MEOX2, EGFR*, and *EYA4* of *C*_2_ have the GO classification “nucleus” and *ARSJ, PIPOX, PLA2G5, POSTN, LAMA2, EGFR,* and *KCNF1* have the classification “extracellular region.” The heat map, shown in Figure 4(b), clearly indicates that genes in *C*_1_ and *C*_2_ provide a significantly better separation of the core members in the two groups.

We conducted the corresponding analysis for the validation data set combined from four public cohorts (see “http://tcga-data.nci.nih.gov/docs/publications/gbm_exp/”) using genes in *C*_1_ and *C*_2_ [1]. We again find a clear difference in fold changes of the genes between the two partitions. In addition, the fractional sizes of the partitions are similar to those of Figure 4(b). (The assignments of the cancer subtype of validation samples are not given at the TCGA site.)

### 3.5. Bisection of the Classical/Mesenchymal Patient Samples

The next step is to partition patient samples in group A, which we found to contain exclusively classical and mesenchymal samples. The computations described above are repeated, however, only using 60 patient samples assigned to the partition. The gene groups for the partition are

(5) C1(CM)={ASCL1,MPPED2,CSPG5,BCAN,DSCAM, DPF1,ZNF606,ZNP30,ZNF419,SGTA, ZNF8,DGKB,EGFR,PHC1,BLM},C2(CM)={TNFAIP8,SLC25A24,OLFML2B,SFT2D2, KYNU,DKK1,RRAS,GUSB,HEPH,ENPP4, NDN,SPAG4,MTAP,KLHL9,COL11A1}.

Genes in *C*_1_^(CM)^ lie in chromosomes 1, 3, 7, 11, 12, 15, 19, and 21. *DPF1, SGTA*, and the four zinc fingers belong to chromosome 19 (19q13) and have the GO classification “intracellular.” They along with *ASCL1*, *BLM*, and *PHC1* have the GO classification “nucleus.” Genes in *C*_2_^(CM)^ belong to chromosomes 1, 2, 5, 6, 7, 10, 15, 19, and the X-chromosome. *TNFAIP8, SLC25A24, KYNU, SPAG4*, and *MTAP* have the classification “cytoplasm.”

**Figure 5:**
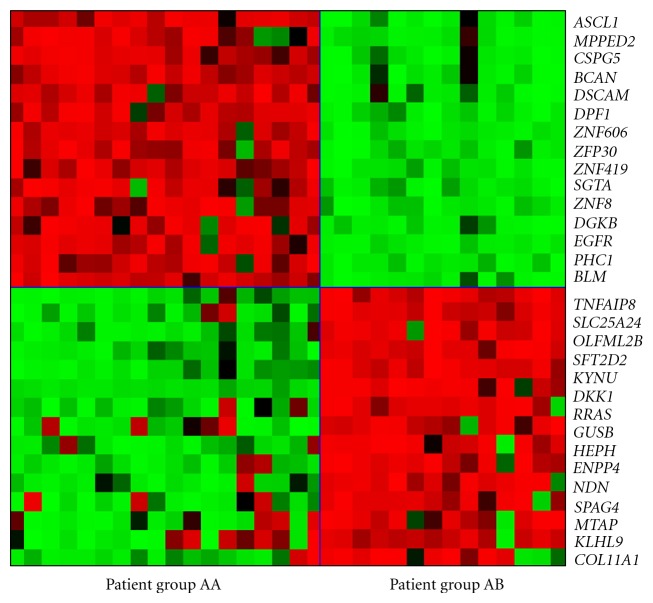
The heat map for the bisection of the classical/mesenchymal group with red (resp., green) representing fold inductions higher (resp., lower) than the mean. The horizontal blue line separates the gene groups *C*_1_^(CM)^ and *C*_2_^(CM)^, while the vertical line partitions the two components of patient samples. Groups AA and AB contain patient samples of the classical and mesenchymal subtypes, respectively.

The first group of patient samples contains 18 members, 17 of which have been assigned to the classical subtype in [1]. The remaining sample has no assignment in TCGA. The second group contains 14 samples, all of which have been assigned the mesenchymal subtype in [1]. The heat map for the bisection of the classical/mesenchymal group is given in Figure 5. The complete list of core patient samples is given in Supplementary Materials (see Supplementary Materials available online at doi:10.5402/2012/381023). A similar bisection of patient group B, which contains the proneural samples, was not possible.

### 3.6. Prediction Model for GBM Subtypes

Fold inductions of genes belonging to *C*_1_, *C*_2_, *C*_1_^(CM)^, and *C*_2_^(CM)^ can be used to predict the membership of a new sample in the three core groups. The choice is based on the mean correlation distance between the patient sample and members of each core group.

The first step is to determine if the new sample belongs to the classical/mesenchymal or proneural core groups. The determination is made using genes belonging to *C*_1_ and *C*_2_ and computing the mean correlation distance between the new sample and those in each partition. However, we must first determine the “radius” (for the correlation distance) within which the membership for each group is assigned. It is chosen as follows. For every patient sample in the classical/mesenchymal core group, we compute its mean correlation distance from the remaining members of the group. The largest of these values is defined to be the radius of the classical/mesenchymal subgroup of patient samples. This choice guarantees that all core patient samples in the group are assigned correctly, and that those outside the core group are not assigned to it. The computed cutoffs for the classical/mesenchymal and proneural core groups are *R*_cm_ = 0.70 and *R*_*p*_ = 0.42, respectively. A new patient sample whose mean correlation distance from members of the classical/mesenchymal core group is smaller than *R*_cm_ is assigned to that group, and any sample closer than *R*_*p*_ to the proneural subgroup is assigned to it.

Next, we use genes in *C*_1_^(CM)^ and *C*_2_^(CM)^ to assign the membership of any patient sample in the classical/mesenchymal core group. The corresponding cutoff radii are *R*_*c*_ = 0.63 and *R*_*m*_ = 0.30. It should be noted that these selection criteria are strict. This ensures proper identification of the subtype. Such accuracy necessarily leaves some samples uncategorized. In such situations, a relaxation of the criteria can allow the subtype to be estimated.  Table 1 shows the mean correlation distances between the test samples and the core groups. Our predictions for membership in the core groups are given in column 6. The last column of the Table gives the assignment of the GBM subtype given by TCGA (see “http://tcga-data.nci.nih.gov/docs/publications/gbm_exp/”) [1]. Five of the test samples did not belong in the core subgroups identified in [1], and our calculation agrees. Of the remaining 15 patient samples, 5 do not belong to any of our three core groups. (In fact, 4 of these have been assigned to the neural subgroup.) Each of the remaining 10 test samples are assigned the subtype of [1].

## 4. Discussion

Robust prognostics for many subtypes of cancer are yet to be discovered [23, 32]. High-throughput genomics offers a possible approach for early and reliable cancer prognosis [8, 38]. Genomic biomarkers may provide an accurate determination of the subtypes of a malignancy.

Clustering algorithms require the definition of a genomic distance between patient samples. Since there is no natural measure, topological approaches to partitioning are likely to prove more robust. Second, although techniques have been proposed to identify the optimal number of partitions of a set of objects, its value can be sensitive to the choice of the gene panel used for profiling. We introduced an algorithm that circumvents these problems. It was used to partition the 202 GBM patient samples whose genetic profiles are given at the publicly accessible TCGA site. We identified a set of genes most useful for the partitioning and to determine core groups of samples for each subtype of GBM.

The selection of the gene set requires several steps. We argued that a necessary condition for the inclusion of a gene was that its fold inductions lie on a distribution with a (relatively) small value of the nondimensionalized standard deviation *σ*, indicating a bimodal distribution. We selected 60 such genes for the remaining analysis. We then used persistent homology to streamline the set further by only retaining genes that belong in groups; genes within a group exhibit highly correlated variations of expression levels between patient samples. We identified two groups of genes *C*_1_′ and *C*_2_′, each containing 15 genes. Many of them do not play a pivotal role in gene regulatory networks associated with GBM [1–4, 22, 23, 39] and may be downstream nodes. It would be interesting to determine if they can be implicated directly in the histopathological criteria used to define the malignancies [23].

Next, we use the differential expression of genes in *C*_1_′ and *C*_2_′ to bisect the patient samples. The two partitions contain 84 and 76 patient samples. Using the heat map for guidance, we selected 60 and 44 samples from the two partitions as its core members. Once the core patient samples in each group are known, it is possible to determine the most suitable gene set to differentiate between the partitions. The new gene groups *C*_1_ and *C*_2_ provide a significantly sharper heat map. We find that the first core group contains patient samples of the classical and mesenchymal subtypes, while the second contains proneural samples.

Our algorithm is “robust” in the following sense. (1) Patient subgroups derived from the analysis depend only weakly on the number of genes used for the persistent homology step (*N* = 60 for the results reported here). For the GBM data, barcodes (Figure 3(a)) for a range of choices for *N* between 50 and 120 are similar, with the component appearing at *r*_*c*_ ≈ 0.6 lasting for a smaller range in cutoff as *N* increases. Values of *N* < 50 do not give uniform results, and those larger than 120 cannot be justified from Figure 2(b). (2) The patient groups are very weakly dependent on the number of genes *N*_*g*_ chosen for the panels *C*_1_ and *C*_2_ (*N*_*g*_ = 15 for each set). We could choose *N*_*g*_ as small as 10 or use all genes in the sets *C*_1_′ and *C*_2_′ for the analysis with similar results.

A unique aspect of our approach is that each subgroup can now be further bisected, albeit using different gene sets. Specifically, the classical/mesenchymal group was subdivided using sets *C*_1_^(CM)^ and *C*_2_^(CM)^, each containing 15 genes. Fold inductions of a panel of 59 genes were needed for both bisections. It is known that that classical patient samples exhibit a high level of expression of *EGFR* [1, 9]. Consistent with this, we find that *EGFR* is over expressed in the classical/mesenchymal partition in Figure 4 and in the classical group in Figure 5. The proneural subtype of GBM is associated with high alterations in *TP53*, *PDGFRA*, and *IDH1*. However, these genes are not found in *C*_1_ or *C*_2_. It is possible that, although there are large alterations in the levels of these genes, the mean values in the ensemble (of proneural samples) do not change significantly. The high levels of mutations of *NF1* of mesenchymal samples are not reflected in the gene sets as well.

We do not find a singe partition for the neural subtype of GBM. Our analysis suggests that patient samples in this group lie, not on a single partition, but on several small partitions. This is consistent with a lack of clear defining genomic characteristics for the subtype [1].

We used the panel of 59 genes in *C*_1_, *C*_2_, *C*_1_^(CM)^, and *C*_2_^(CM)^ to introduce a prediction model for the subtypes of GBM. The test involved the computation of the mean correlation distance between a new sample and members of each core group. Fifteen out of the 20 test sample predictions agreed with the results from the clustering calculation. Of these, 5 were unassigned by both algorithms. Four of the remaining samples were categorized as neural by the clustering method but were unassigned by ours since the neural group was not found to be a single cluster.

Of the genes in *C*_1_, *C*_2_, *C*_1_^(CM)^, *C*_2_^(CM)^, only *EGFR* has been previously implicated in GBM. However, other gene groups may have functions related to cancer (the functional annotations can be found in “GeneCards,” the human Gene Compendium, Weizmann Institute of Science). One example is a group related to intercellular scaffolding: *DCX* translates to a microtubule-associated protein, *DNM3* is involved in producing microtubule bundles, *POSTN* plays a role in extracellular matrix mineralization, *RRAS* regulates the actin cytoskeleton, and *COLL11A1* may play a role in fibrillogenesis. Another group is known to be expressed in brain function: *RAB33A* is a member of the Ras oncogene superfamily, *GPR17* may mediate in brain damage, and *NR2E1* may be required for brain development. A third group of genes is associated with differentiation, development, and the cell cycle: they include *EDHB4*, *ASCL1* involved in early stages of development, *MPPED2*, which plays a role in the development of the nervous system, *CSPG4*, which functions as a growth and differentiation factor, *BCAN* involved in terminal differentiation, *MEOX2*, which plays a regulatory role in muscle cells, and *NDN*, which is a growth suppressor involved in cell cycle arrest. The zinc fingers *ZFP30, ZNF419, ZNF8*, and *ZNF606* are involved in transcriptional regulation.

## 5. Conclusions

We have introduced a novel algorithm for genomic subtyping of cancer samples. Unlike prior clustering techniques, we predetermine a compact set of genes to be used in the analysis through the nondimensionalized standard deviation and persistent homology. We next use the gene set to bisect the patient samples using persistent homology. Importantly, the gene set selected for the analysis depends on the group of patient samples to be analyzed. Hence, if needed, partitions obtained from the first step can be further bisected. Our approach not only provides a robust partitioning of patient samples, but can also be used to identify a panel of genes to be used as a biomarker. The gene panels have been used to introduce a prediction model to determine if a new patient sample belongs to the core subgroups associated with classical, mesenchymal, and proneural subtypes of GBM.

## Supplementary Material

The core patient samples in the proneural, classical, and mesenchymal subtypes of glioblastoma multiforme. Here, we have used the nomenclature for patient samples as given by TCGA. Our assignments of the samples shown in red are different from those of TCGA. The samples highlighted in green have not been provided an assignment by TCGA.

## Figures and Tables

**Table 1:** The mean correlation distance between each test sample and the core samples of the classical, mesenchymal, and proneural subtypes of GBM. The sample is predicted to belong to a subtype if its mean correlation distance to the core group is smaller than a predetermined value. The 10 assignments provided by our prediction model agree with the TCGA classification.

Patient sample	Correlation distance (Clas/Mes)	Correlation distance (Proneural)	Correlation distance (Classical)	Correlation distance (Mesenchymal)	Prediction	TCGA Assignment
TCGA-02-0015	0.60 ± 0.18	1.46 ± 0.11	1.04 ± 0.23	0.72 ± 0.14		
TCGA-02-0021	0.39 ± 0.25	1.78 ± 0.07	0.31 ± 0.13	1.61 ± 0.06	C	C
TCGA-02-0026	1.62 ± 0.17	0.27 ± 0.12	—	—	P	P
TCGA-02-0037	0.56 ± 0.15	1.52 ± 0.08	0.88 ± 0.21	1.03 ± 0.11		
TCGA-02-0051	0.57 ± 0.27	1.39 ± 0.13	1.26 ± 0.14	0.28 ± 0.08	M	M
TCGA-02-0058	1.49 ± 0.20	0.45 ± 0.17	—	—		
TCGA-02-0074	1.58 ± 0.18	0.25 ± 0.13	—	—	P	P
TCGA-02-0075	0.35 ± 0.19	1.74 ± 0.11	1.23 ± 0.14	0.36 ± 0.11	M	M
TCGA-02-0106	0.38 ± 0.17	1.73 ± 0.08	0.91 ± 0.14	0.56 ± 0.12		M
TCGA-02-0451	0.73 ± 0.24	1.38 ± 0.13	—	—		N
TCGA-06-0168	1.19 ± 0.15	0.87 ± 0.11	—	—		
TCGA-06-0171	1.48 ± 0.13	0.47 ± 0.08	—	—		N
TCGA-06-0185	0.84 ± 0.33	1.31 ± 0.17	—	—		N
TCGA-06-0190	0.49 ± 0.25	1.56 ± 0.16	1.54 ± 0.14	0.35 ± 0.10	M	M
TCGA-06-0410	1.41 ± 0.28	0.41 ± 0.15	—	—	P	P
TCGA-06-0413	1.67 ± 0.18	0.17 ± 0.08	—	—	P	P
TCGA-08-0359	1.64 ± 0.14	0.25 ± 0.08	—	—	P	P
TCGA-08-0380	1.09 ± 0.24	0.91 ± 0.19	—	—		N
TCGA-08-0517	1.50 ± 0.17	0.38 ± 0.11	—	—	P	P
TCGA-08-0521	0.56 ± 0.25	1.47 ± 0.15	1.58 ± 0.09	0.38 ± 0.13		
